# Divergent Human Papillomavirus Genotype Spectra in Three Indigenous Communities of Venezuela: Implications for Vaccine Choice and Cervical Screening

**DOI:** 10.3390/diseases14100384

**Published:** 2026-10-19

**Authors:** María Fátima Garcés, Jefferson Guzmán Silva, Daniel Aranguren, Lina Figueira, Ricardo Blanch, Marwan Said Aguilar Mejía, Yacelli Bustamante Siberio, Juan Bautista De Sanctis, Alexis Hipólito García

**Affiliations:** 1 Escuela de Bioanálisis “Dr. Rafael Rangel”, Faculty of Medicine, Universidad Central de Venezuela, Caracas 1040, Venezuela; mariafatimagarcesdasilva@gmail.com (M.F.G.); jeffguzman21@gmail.com (J.G.S.); yacelli@gmail.com (Y.B.S.); 2 Escuela de Medicina “Luis Razetti”, Faculty of Medicine, Universidad Central de Venezuela, Caracas 1040, Venezuela; daniel_aranguren@outlook.com; 3 Gynecology Department, Hospital Universitario de Caracas, Escuela de Medicina “Luis Razetti”, Faculty of Medicine, Universidad Central de Venezuela, Caracas 1040, Venezuela; figueiralina@gmail.com (L.F.); rebc48@yahoo.com (R.B.); 4 Instituto Anatomopatológico “Dr. José Antonio O’Daly”, Faculty of Medicine, Universidad Central de Venezuela, Caracas 1040, Venezuela; marwaguilar@gmail.com; 5 Instituto Nacional de Higiene “Rafael Rangel”, Ministry of Popular Power for Health, Caracas 1050, Venezuela; 6 Institute of Molecular and Translational Medicine, Faculty of Medicine and Dentistry, Palacký University, 779 00 Olomouc, Czech Republic; juanbautista.desanctis@upol.cz; 7 Instituto de Inmunología “Dr. Nicolás Enrique Bianco C.”, Faculty of Medicine, Universidad Central de Venezuela, Caracas 1040, Venezuela

**Keywords:** human papillomavirus, genotyping, indigenous population, Venezuela, Wayuu, Eñepa, Warao, cervical cytology, HPV vaccine, health disparities

## Abstract

**Background**: Genotype-resolved data on human papillomavirus (HPV) are almost absent for Venezuelan indigenous women. **Methods**: We studied 260 women from La Guajira (Wayuu, *n* = 94), Maniapure (Eñepa, *n* = 141), and Delta Amacuro (Warao, *n* = 25), processing all self-collected samples with a single real-time PCR assay that resolves 21 genotypes; cytology was available for 144. **Results**: HPV prevalence was 55.4% (95% CI 49.3–61.3) and high-risk prevalence was 51.2% (95% CI 45.1–57.2), rising from 42.6% in La Guajira to 59.6% in Maniapure and 80.0% in Delta Amacuro (*p* = 0.001). Nineteen genotypes were detected. HPV-31 was most frequent (15.8%), followed by HPV-16 (11.5%), and each community had its own profile, with HPV-68 and HPV-56 leading in Delta Amacuro. Half of the infected women carried more than one type. Cytology and HPV testing overlapped little: 20 of 78 HPV-positive women with a smear (25.6%) had an abnormal result (κ = 0.056). At least one nonavalent vaccine type was present in 69.4% of infected women, compared with 30.6% for the bivalent vaccine. **Conclusions**: HPV vaccination is not part of the Venezuelan national program; these data favor the nonavalent formulation, and HPV-based screening would need a feasible triage pathway for the many women who test positive.

## 1. Introduction

Human papillomavirus (HPV) is the most common sexually transmitted infection; 75–80% of sexually active people acquire it at some point in their lives [1]. Persistent infection with high-risk genotypes is the necessary cause of cervical cancer and contributes to other anogenital and oropharyngeal cancers [2,3]. Prevalence among women with normal cytology is 11.7% worldwide and 16.1% in Latin America and the Caribbean [4], and the burden of cervical cancer falls mostly on countries with lower human development [5].

Venezuelan data on HPV are fragmented and come mostly from single-center studies [6,7]. Because national cancer statistics are not published regularly, the best available figures are the ICO/IARC estimates: about 3709 new cases and 2129 deaths a year, an age-standardized incidence of 22.2 per 100,000 women, with cervical cancer the leading cause of cancer death among women aged 15 to 44 years [8]. Reported HPV prevalence depends on the population and the method, from 12.5–15.6% with hybrid capture in asymptomatic urban women [9,10] to 34–91% with PCR in women with clinical or cytological suspicion [11,12,13,14,15,16,17,18,19,20,21,22,23]. Larger national and regional series fall within the same range [24,25,26,27].

Indigenous women have received some attention. An early survey with a seven-type probe panel found no infection among 46 Hüottüja (Piaroa) women [28]. Across Latin America, cervical cancer is the leading cancer among indigenous women and is diagnosed late, and some groups carry very high high-risk HPV prevalence [29,30]. In Venezuela, HPV was found in 35.1% of indigenous women in Amazonas State, with a heavier oncogenic burden than in urban mestizo women [31,32], and in 45.7% of Eñepa women against 20.0% of Creole women attending the same clinic in Maniapure [33]. In Zulia State, cytological alterations were found in two-thirds of Barí, Yukpa and Wayuu women, and Wayuu women face marked cultural barriers to screening [34,35,36]. In all these settings, isolation and scarce diagnostic services delay detection [29,30,33].

Most of this literature relies on assays that pool oncogenic types or resolve only HPV-16 and HPV-18, although genotype resolution is what decides which vaccine would protect the most women [3,37]. An earlier analysis of the La Guajira and Maniapure samples used a different platform at each site and could not compare genotypes between communities [37,38]. In the present study, all samples from three communities were processed with one protocol that resolves 21 genotypes. We aimed to estimate HPV and high-risk HPV prevalence, describe genotypes and coinfection by community, describe how cytology and HPV DNA detection relate, and estimate the share of infections targeted by current vaccines.

## 2. Materials and Methods

### 2.1. Study Design and Setting

This multi-site cross-sectional study was carried out in three indigenous communities of Venezuela: La Guajira (Zulia State, Wayuu), Maniapure (Bolívar State, Eñepa) and Delta Amacuro (Delta Amacuro State, Warao) (Figure 1). Women were enrolled in four field campaigns between February 2024 and June 2025: La Guajira in February 2024, Maniapure in June 2024 and June 2025, and Delta Amacuro in April–May 2025. Sampling took place during the medical outreach trips of the Campamento Universitario Multidisciplinario de Investigación y Servicio (CUMIS), the extension program of the Universidad Central de Venezuela, which offer general care to the whole community; HPV testing was one of several services and was announced by an open call. La Guajira and Maniapure can be reached by road, and both communities deal regularly with the non-indigenous (*criollo*) population. The Warao of Delta Amacuro live scattered along the river channels (*caños*) of the Orinoco Delta and can only be reached by boat; most participants from this site lived near Tucupita, the state capital. As far as we know, no HPV study had been carried out before among Warao women in Delta Amacuro. Earlier work in this research line used different molecular platforms at each site [33,37,39]; here, all samples were processed under a single protocol, and the present dataset supersedes the earlier partial analysis [38].

### 2.2. Study Population

Eligible participants were sexually active women living in the participating communities, with or without earlier clinical or cytological findings suggestive of HPV infection. Women were not selected on the basis of symptoms or earlier results, because the aim was to describe infection in the community as a whole. Eligibility was defined by sexual activity rather than age, because sexual debut occurs early in several of these communities. Women with active menstrual bleeding, confirmed or suspected pregnancy or a contraindication to sampling were excluded. Participation was voluntary. In Maniapure, the team also invited women at schools, traditional dwellings and the local health center; in Delta Amacuro and La Guajira, care was provided at municipal schools. The approximate response rate was 90% in La Guajira and Maniapure and about 50% in Delta Amacuro, where women from settlements up to six hours away by boat could rarely reach the field site. Other reasons for non-participation were work commitments and an isolated settlement in La Guajira and, in Maniapure, partners who did not allow women to attend a gynecological consultation. In total, 260 women were included: 94 from La Guajira, 141 from Maniapure and 25 from Delta Amacuro.

### 2.3. Ethical Considerations

The study followed the Declaration of Helsinki (2013 revision) and Venezuelan regulations on human research. The protocol was approved by the Bioethics Commission (Comisión de Bioética) of the Escuela de Medicina “Luis Razetti”, Facultad de Medicina, Universidad Central de Venezuela (protocol CBI-EMLR-58-2024, 17 January 2024), before the first field campaign. Nine participants were under 18 years of age, the youngest being 13; for each, the participant gave written assent and a legal guardian gave written consent on the combined consent–assent form approved by the Committee. Before enrolment, participants received a culturally appropriate explanation of the study in Spanish, with community interpreters where needed, and gave written informed consent; participants who could not write provided a fingerprint in the presence of a witness. We coded samples anonymously and returned individual results to support clinical referral. The Ethics Committee approved the manuscript submission.

### 2.4. Sample Collection

After a short session on the technique, each woman self-collected a vaginal sample with a sterile Dacron swab rotated three to four times. Swabs were placed in 15 mL tubes containing 1 mL of nucleic-acid-stabilizing lysis buffer, transported under refrigeration to the Laboratorio de Investigaciones Básicas y Aplicadas, Escuela de Bioanálisis, Universidad Central de Venezuela, and stored at −20 °C as they arrived. We extracted DNA in batches, one community at a time, 1 to 3 months after collection. Media of this type protect DNA independently of refrigeration, with almost perfect agreement (κ = 0.98) between testing within six days and after one month of ambient storage [40]. Where feasible, the attending gynecologist also took a conventional cervical smear.

### 2.5. Cytological Evaluation

The two gynecologists on the team (R.B. and L.F.) took conventional (Papanicolaou) smears with an Ayre spatula. Those from La Guajira and Maniapure were read at the Laboratorio de Patología San Román and those from Delta Amacuro were read at the Laboratorio Biotecnovid; the two laboratories were in constant communication throughout the study and coordinated their reporting. They were reported with the 2014 Bethesda terminology as negative for intraepithelial lesion or malignancy (NILM), low-grade squamous intraepithelial lesion (LSIL) or high-grade squamous intraepithelial lesion (HSIL). Inflammatory and infectious changes fall within NILM and were only described. Cytology was available for 144 of the 260 women (55.4%); in the others, a smear could not be obtained or was inadequate.

### 2.6. DNA Extraction and Genotyping

DNA was extracted with a modified Bunce precipitation method [41], and its integrity was checked on 0.75% agarose gels. Genotyping was performed by real-time PCR on a Bio-Rad CFX Opus 96 system (Bio-Rad Laboratories, Hercules, CA, USA) with the HPV Genotypes 21 Real-TM Quant kit (Sacace Biotechnologies, Como, Italy; REF V21-100 FRT; CE-IVD), which resolves 21 types individually in eight reaction tubes per sample. Every tube carries an internal control, and the eighth also a human DNA sample intake control; the analytical limit of detection is 5 copies per reaction. Assay design, positivity criteria, analytical performance and the contamination controls (separate pre- and post-PCR areas, blank extraction and no-template controls in every run, and every sample tested in two separate PCR runs) are described in Supplementary Methods S1. All samples amplified a valid internal control, confirming adequate material free of PCR inhibition [42].

High-risk types were the 14 used in commercial assays and WHO guidance: HPV-16, 18, 31, 33, 35, 39, 45, 51, 52, 56, 58, 59, 66 and 68. IARC classifies twelve of them as carcinogenic (group 1), HPV-68 as probably carcinogenic, and HPV-66 as possibly carcinogenic [43]. The 2026 WHO guideline sorts the twelve group 1 types into four management groups: 1a (HPV-16), 1b (HPV-18 and 45), 1c (HPV-31, 33, 35, 52 and 58) and 1d (HPV-39, 51, 56 and 59) [44]. The remaining types were grouped as low-risk (HPV-6, 11 and 44) or possibly carcinogenic (HPV-26, 53, 73 and 82) [43]. As a sensitivity analysis, high-risk prevalence was also calculated with the 12 group 1 types alone and with the manufacturer’s broader definition. A sample was HPV-positive when any genotype was detected and high-risk-positive when any of the 14 high-risk types was present.

### 2.7. Statistical Analysis

Age was not normally distributed (Shapiro–Wilk test) and was compared with the Kruskal–Wallis test, with Dunn post hoc comparisons and Bonferroni correction, as well as with the Mann–Whitney U test. Categorical variables were compared with the chi-square test or Fisher’s exact test, the latter with simulated *p*-values (B = 10,000) when expected counts were small. Proportions are given with 95% Wilson confidence intervals, which behave better than Wald intervals in small samples such as Delta Amacuro. Agreement between cytology and qPCR was described with Cohen’s κ, interpreted according to Landis and Koch [45]. Without histology, neither test could serve as the reference, so no sensitivity, specificity, or predictive values were calculated. Genotypes were also described by age group (<18, 18–24, 25–30, 31–40, 41–50 and >50 years), by cytological category and by WHO 2026 risk group [44]. A multiple correspondence analysis (MCA) of origin, age group, cytological category and HPV status in the 144 women with complete data was used as an exploratory, descriptive tool to show how infection patterns differ between communities; it was not used to test hypotheses. A two-tailed *p* < 0.05 was considered significant. Analyses were performed in R version 4.5.2 [46] with ggplot2 v4.0.1 [47], tableone v0.13.2 [48], FactoMineR v2.13 [49] and dunn.test v1.3.6 [50]. The map in Figure 1 was drawn from GADM version 4.1 boundaries [51], retrieved with the geodata package [52], with ggrepel [53] and ggspatial [54].

## 3. Results

### 3.1. General Characteristics

The 260 women had a mean age of 34.0 ± 12.3 years (median 32, range 13–76). Age differed between communities (Kruskal–Wallis H = 27.44, df = 2, *p* < 0.001), and all pairs differed: La Guajira 37.7 ± 11.8, Maniapure 33.0 ± 12.4 and Delta Amacuro 25.6 ± 7.3 years. HPV-positive women were younger than HPV-negative women (31.8 ± 12.5 vs. 36.7 ± 11.4 years; Mann–Whitney *p* < 0.001) (Supplementary Tables S1 and S2). Of the 144 women with cytology, 111 presented NILM, 31 had an LSIL, and 2 had an HSIL (Table 1). Within NILM, inflammatory changes were reported in 8 of 14 smears from Delta Amacuro (57.1%), 21 of 65 from Maniapure (32.3%), and none of 32 from La Guajira, and infectious changes were reported in 5 (35.7%), 5 (7.7%), and none. Inflammatory changes appeared in 17.9% of HPV-positive and 22.7% of HPV-negative women, and infectious changes appeared in 9.0% and 4.5% (Supplementary Tables S4 and S5).

### 3.2. HPV and High-Risk HPV Prevalence

HPV prevalence was 55.4% (95% CI 49.3–61.3) and high-risk prevalence was 51.2% (95% CI 45.1–57.2) (Table 2; Supplementary Figure S1). Both differed between communities (χ^2^ = 13.40 and 13.98, df = 2; *p* ≤ 0.001), from 42.6% in La Guajira to 59.6% in Maniapure and 80.0% in Delta Amacuro (Supplementary Table S3). Of the 144 infected women, 133 (92.4%) carried at least one high-risk type. The definition of high risk changed little: prevalence was 48.8% with the 12 IARC group 1 types, 51.2% with the study definition and 53.5% with the manufacturer’s definition, and the ranking of the communities was the same under all three (Supplementary Table S6). Prevalence was highest at 18–24 years (75.9%), lowest at 41–50 years (24.4%) and rose again after 50 (54.2%, *n* = 24); in Delta Amacuro, it stayed at 80% or more from 18 to 40 years (Supplementary Table S7, Figure S2).

### 3.3. Genotype Distribution and Coinfection

Nineteen of the 21 genotypes were detected; HPV-11 and HPV-26 were not found. HPV-31 was the most frequent (41 women, 15.8%), followed by HPV-16 (11.5%), HPV-18 (9.2%), HPV-56 (8.5%), and HPV-39 (8.1%) (Figure 2). Each community had its own profile (Figure 3; Supplementary Table S9): HPV-68 (48.0%), HPV-56 (40.0%), HPV-31 and HPV-16 (36.0% each) in Delta Amacuro; HPV-31 (20.6%), HPV-16 (12.1%) and HPV-39 (11.3%) in Maniapure; and HPV-51 (9.6%), HPV-18 (8.5%) and HPV-45 (6.4%) in La Guajira, where HPV-16 appeared in only 4.3% of women. HPV-31 led in every age group from 18 to 50 years (Supplementary Table S8). Assigned to the highest-risk WHO 2026 group [44] among their genotypes, 20.8% of infected women fell in group 1a, 18.1% in 1b, 27.1% in 1c and 22.2% in 1d, and 11.8% carried only types outside these groups. In Delta Amacuro, 70.0% carried a group 1a or 1b type, and none carried only types outside the groups (Supplementary Table S10, Figure S3).

More than one genotype was found in 74 infected women (51.4%), and 19 (13.2%) carried four or more, up to seven. Coinfection was most intense in Delta Amacuro, with a mean of 3.0 genotypes per infected woman and 35.0% carrying four or more, against 1.9 in Maniapure and 1.8 in La Guajira (Kruskal–Wallis H = 13.87, df = 2, *p* < 0.001; Supplementary Figure S4).

### 3.4. Cytology and HPV DNA Detection

Both results were available for 144 women. An abnormal smear (LSIL or HSIL) was found in 20 of 78 HPV-positive women (25.6%) and in 13 of 66 HPV-negative women (19.7%); agreement was slight (κ = 0.056) and fell below chance in Delta Amacuro (κ = −0.219) (Supplementary Table S11). HPV was detected in 58 of 111 women testing NILM (52.3%), 20 of 31 with an LSIL (64.5%; Fisher’s exact test, *p* = 0.31), and in neither of the two women with an HSIL, both from La Guajira (Table 3). The genotypes found in LSIL samples largely matched those in NILM samples. HPV-31 was about twice as common in the LSIL group (25.8% vs. 12.6%; *p* = 0.09), and no genotype differed significantly between the two categories.

### 3.5. Genotypes Targeted by Licensed Vaccines

Among the 144 infected women, at least one type targeted by the bivalent vaccine was found in 30.6%, by the quadrivalent one in 36.8% and by the nonavalent one in 69.4% (Table 4; Supplementary Figure S5). The nonavalent gain came mainly from HPV-31, 45, 52 and 58 and was largest in Delta Amacuro, from 50.0% to 90.0%. These are woman-level figures. Per detection, nonavalent types accounted for 52.7% (95% CI 47.0–58.4) of the 292 genotypes found and 56.5% (50.3–62.4) of the 255 high-risk ones; in Delta Amacuro, only 48.3% of detections were nonavalent types, because HPV-68 and HPV-56 were so frequent. These infection-level intervals ignore clustering within women and are approximate (Supplementary Table S12). Overall, 30.6% of infected women carried no type included in any licensed vaccine, most often HPV-56, 39, 68, 51 or 59.

### 3.6. Multiple Correspondence Analysis

In the MCA (Figure 4; Supplementary Tables S13 and S14, Figure S6), the first two dimensions explained 29.5% of total inertia. The first (16.6%) separated Delta Amacuro from La Guajira (contributions 19.9% and 19.2%), with Maniapure near the center (0.6%); the 18–24 age group and HPV-positive women lay on the Delta Amacuro side, and the 41–50 group and HPV-negative women lay on the La Guajira side. The second (12.9%) was defined by cytology, with LSIL (contribution 39.9%) opposite NILM. HSIL lay far out on the first dimension but rests on two women.

## 4. Discussion

To our knowledge, this is the first genotype-resolved comparison of HPV infection across three indigenous communities of Venezuela under a single molecular protocol. Infection was common, at 55.4% overall and 80.0% in Delta Amacuro; HPV-31 rather than HPV-16 led the spectrum; and the three communities differed sharply. These figures exceed the global and Latin American estimates for women with normal cytology [4] and agree with what is known of indigenous women in the region [29,30] and in Venezuela, where every matched comparison shows a heavier burden in indigenous than in non-indigenous women [31,32,33,34,35,36].

Several factors may act together. Eligibility admitted women with earlier findings suggestive of HPV, and attendance was voluntary, so infected women may be over-represented. A self-collected vaginal sample tested for 21 individual types detects more infections, including low-load and multiple ones, than cervical sampling with hybrid capture, which explains part of the gap with earlier Venezuelan figures. Sexual debut and marriage occur early in some of these groups, though we did not record sexual histories. Host immunity may also matter: HIV has been documented among the Warao [55], and undernutrition and chronic parasitic infection are common in these territories [56]; both could slow viral clearance, which would fit the heavy coinfection in Delta Amacuro. Infectious changes were somewhat more frequent in HPV-positive women (9.0% vs. 4.5%), although the numbers are small. Finally, none of the communities has organized screening, and an HPV vaccine is not part of the national immunization program; it is available only privately, at the patient’s own expense [8], and is practically out of reach for indigenous women in remote areas.

The three populations are not interchangeable. They differ in age structure, prevalence, coinfection and genotypes, and the MCA places geographic origin as the main axis of variation. The La Guajira profile, led by HPV-51 and HPV-18, reproduces what an independent nested multiplex PCR found in the same community [38]. Elsewhere in the region, the picture is just as varied: HPV-16 led among indigenous women in the Colombian Amazon and in northern Argentina, whereas HPV-58 led in northwestern Ecuador [57,58,59]. Genotype spectra cannot be extrapolated across indigenous populations, and treating “indigenous women” as a single category hides the variation local programs need. In Delta Amacuro, infected women carried three genotypes on average, which fits high transmission in a young population with almost no screening. The sample is small and comes mostly from women living near Tucupita; women from remote channels, with even less access to care, are missing, so these figures offer a first look at an unstudied population.

Host genetics may also contribute. The Warao have a narrow HLA repertoire, consistent with long isolation and endogamy [60]; family studies found only four DR specificities, of which DRw16, the serological equivalent of HLA-DRB1*16, reached a gene frequency of 0.42 [61]. DRB1*1602 has been associated with cervical HPV infection in Andean Bolivian women [62], and HLA class II associations with HPV-related disease are often type-specific [63]. A restricted repertoire could influence which genotypes persist. This remains a hypothesis, since we did not type HLA, and data for the other two communities are scarce: only HLA-DPB1 has been typed in the Wayuu, on the Colombian side of the Guajira [64], and we found no HLA study of the Eñepa. Joint HLA and HPV genotyping in these communities would be a natural next step.

Cytology and HPV DNA testing rarely identified the same women (κ = 0.056). This says little about cytology itself: the two tests look for different things, most infections clear without cellular change, and here they were run on different specimens, a self-collected vaginal swab and a cervical smear taken by the gynecologist. The two HSIL cases, both HPV-negative, illustrate the point; without biopsy we cannot tell whether they were false-positive smears, lesions caused by types outside the panel or sampling failures. Compared with histology, conventional cytology detects CIN2+ with a pooled sensitivity of 62.5% and HPV testing with 89.9% [65], and our figures are not comparable. Our data show practical limits: only 55.4% of women could provide a smear, whereas self-collection performs as well as clinician sampling for PCR-based tests [66]. Self-collected HPV testing is the most workable primary screening option for these communities, as the WHO recommends [44].

With about half of the women testing positive, the question is what comes next. The 2026 WHO guideline [44] uses limited or extended genotyping as a built-in triage step, adapted to follow-up capacity: where at least 60% of women can be followed up, it suggests treating groups 1a to 1c and triaging group 1d, and where follow-up is weaker, it suggests treating every eligible woman with a carcinogenic type [44]. These communities fall below that threshold, particularly Delta Amacuro. In our data, 38.9% of infected women were in groups 1a or 1b and 27.1% in 1c, so a scheme that treated only 1a and 1b would send more than a quarter of them to triage, including most HPV-31 infections. In Delta Amacuro, coinfection placed every infected woman in a WHO group, and 70.0% had HPV-16, 18 or 45. A realistic pathway would combine self-collected HPV testing, genotype-based prioritization and visual or cytological triage, and treatment on the same trip whenever possible.

For vaccination, a bivalent program would target a detected genotype in fewer than one in three infected women, and the nonavalent vaccine in seven in ten, rising to nine in ten in Delta Amacuro. HPV-11 was in the panel and never found, so these figures are complete. They describe how closely current genotypes match each vaccine, not vaccine effectiveness, since vaccines prevent future infections; what they indicate is which formulation would best protect the next generation. Attribution data agree: HPV-16 and 18 cause about 70% of cervical cancers worldwide, and the nonavalent types cause close to 90% [67]. Because the HPV vaccine is not yet in the Venezuelan schedule, this choice is still open. Even so, 30.6% of infected women carried only types outside every licensed vaccine, so vaccination cannot replace screening in these communities.

Age and structural conditions also shape these results. Infected women were about five years younger than uninfected women, as expected from transient infection in the young, but this does not mean low risk later in life: both HSIL cases occurred in La Guajira, the oldest community, and prevalence rose again after 50. All three communities are hard to reach: Maniapure by unpaved roads [33], the Warao only by boat, with journeys of six hours or more [68], and the Wayuu across an arid border region with thin health coverage [34]. Opportunistic screening, early sexual debut and the deterioration of health services in remote territories [68,69] point to structural rather than biological drivers, which molecular tests alone will not resolve [30,69].

## 5. Limitations

This study has limitations. The cross-sectional design cannot separate transient from persistent infection. No sample size was calculated a priori: this was an exploratory survey whose size was set by the women who could be reached during the field trips, and the Delta Amacuro estimates, based on 25 women, are imprecise and hypothesis-generating. Cytology was available for 144 women; those with and without it were similar in age and HPV positivity (*p* = 0.90 and *p* = 0.75), but availability ranged from 92.0% in Delta Amacuro to 43.6% in La Guajira (*p* < 0.001), so agreement figures apply to that subset. Genotypes were not confirmed by sequencing, histology was unavailable, and self-collected samples may differ in adequacy from clinician-collected ones [66,70]. We did not record sexual history, vaccination status, HIV serology or HLA type, which rules out multivariable adjustment. Age, the main measured confounder, differed between communities, so part of the differences between sites may reflect age structure. Participation was voluntary, most Delta Amacuro participants lived near Tucupita, and eligibility admitted women with earlier findings suggestive of HPV, all of which may affect representativeness. Smears from Delta Amacuro were read in a different laboratory than those from the other two communities, so we cannot exclude differences in reporting practice.

## 6. Conclusions

HPV infection affects more than half of the indigenous women studied in these three Venezuelan communities and four in five in Delta Amacuro, and nearly all infections involve high-risk types. HPV-31, not HPV-16, leads the spectrum, which differs from one community to the next. The nonavalent vaccine would target a detected genotype in seven of ten infected women, against three in ten for the bivalent vaccine; if HPV vaccination enters the national program, these data favor the nonavalent formulation. Cytology and HPV testing measure different things and seldom agreed. Self-collected HPV testing is feasible in these settings but will need a clear triage pathway, for which the 2026 WHO genotype groups [44] offer a starting point. Vaccine choice for indigenous territories should rest on local genotype data, starting with Delta Amacuro.

## Figures and Tables

**Figure 1:**
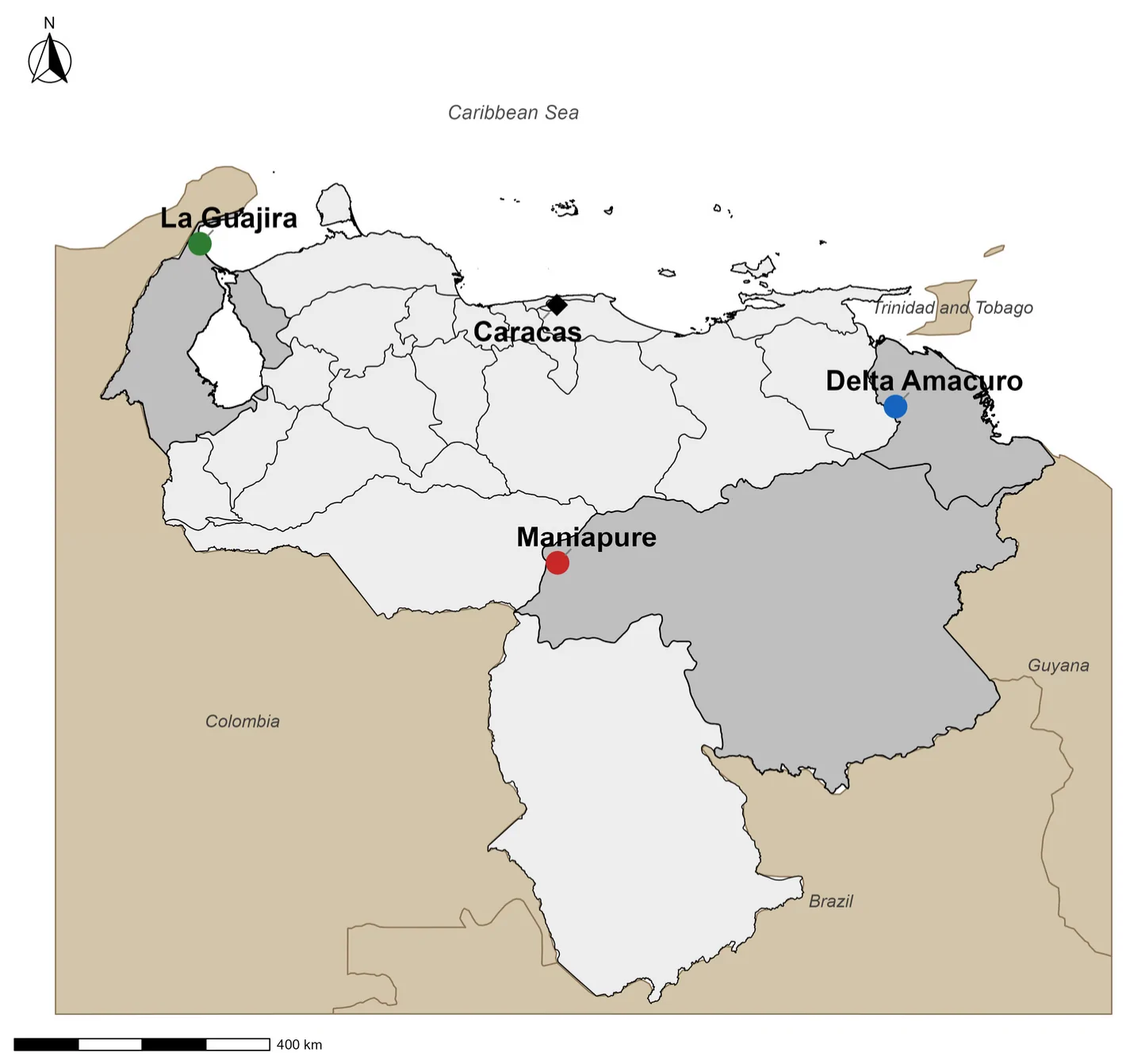
Geographic location of the three indigenous communities included in this study, relative to Caracas: La Guajira (Zulia State, Wayuu), Maniapure (Bolívar State, Eñepa) and Delta Amacuro (Delta Amacuro State, Warao). Shaded states indicate the sampling regions. Map generated in R; see Section 2.7 for data sources and packages.

**Figure 2:**
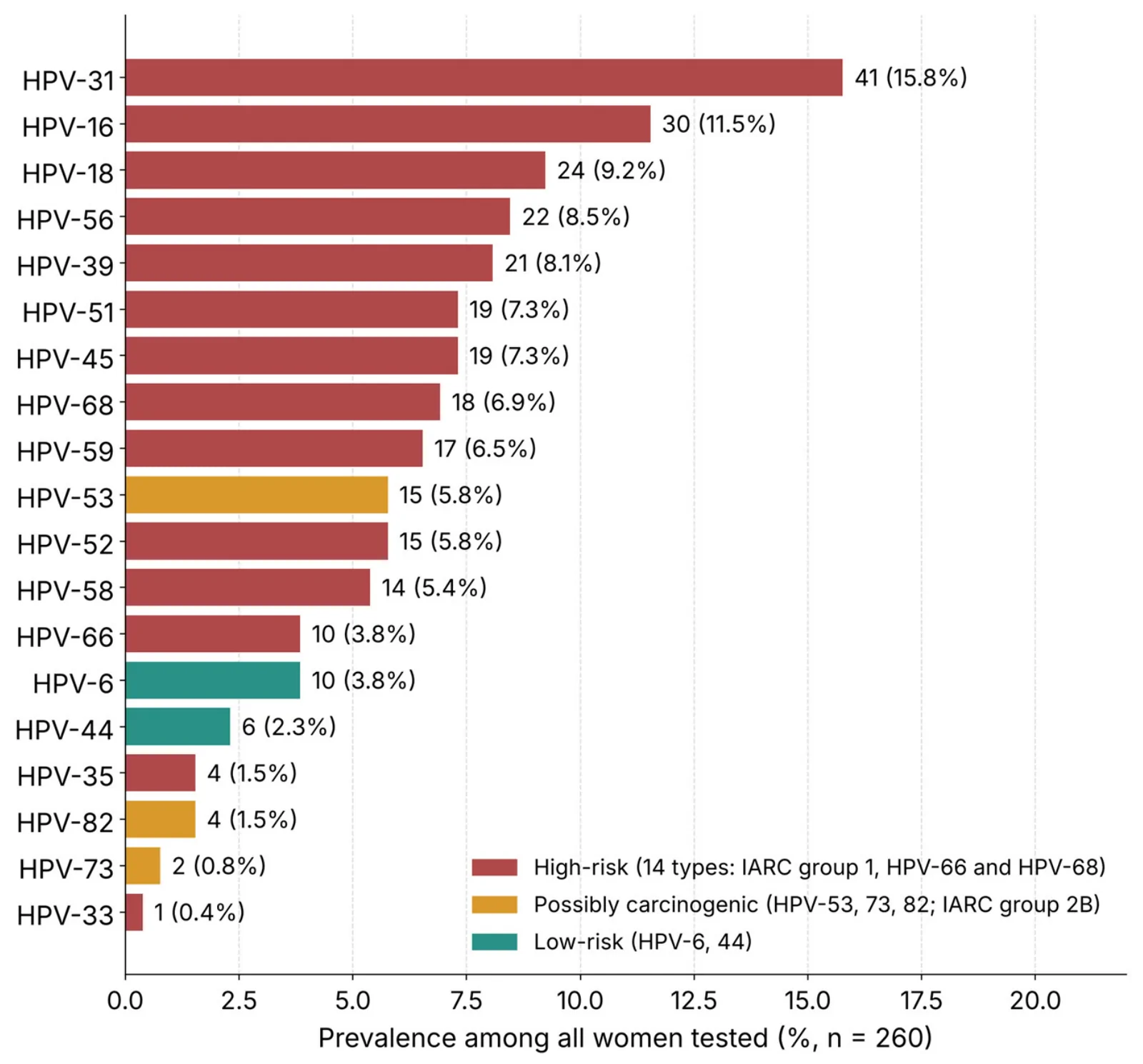
Frequency of the 19 HPV genotypes detected in the whole cohort (*n* = 260), expressed as the percentage of women tested in whom each genotype was identified. Bars are colored by risk category: high-risk (the 14 types defined in Section 2.6), possibly carcinogenic (IARC group 2B) and low-risk.

**Figure 3:**
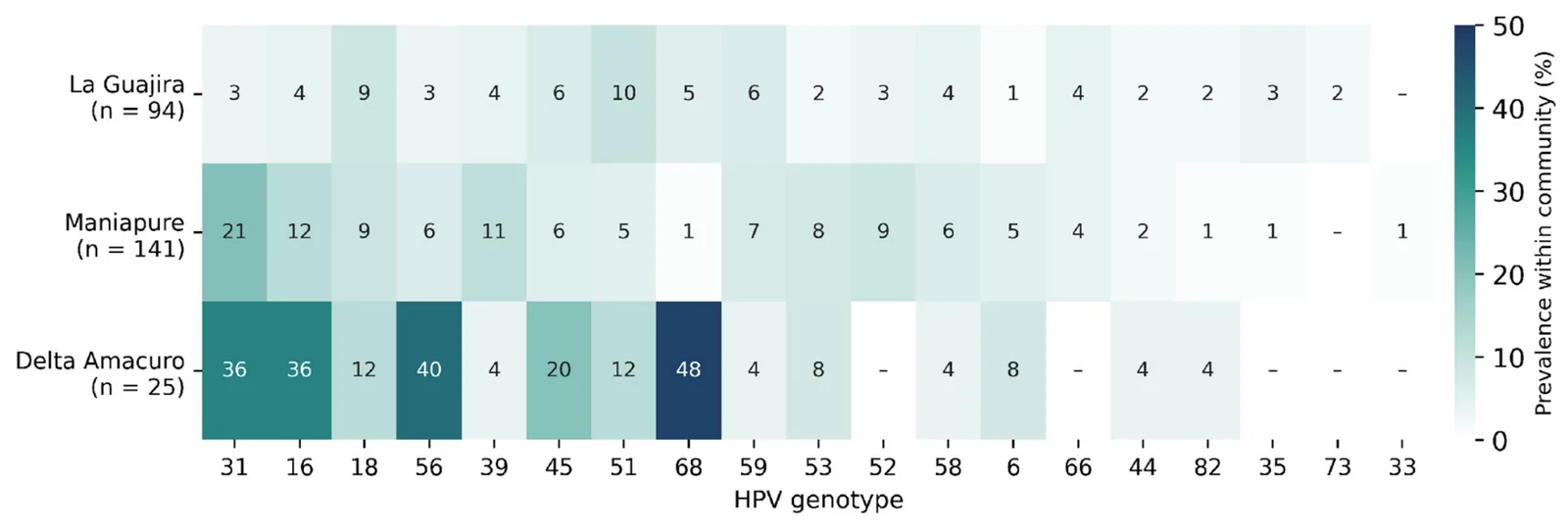
Heat map of genotype-specific prevalence within each community, with genotypes ordered by overall frequency in the cohort. Cell values are percentages of women tested at each site; a dash indicates that the genotype was not detected in that community.

**Figure 4:**
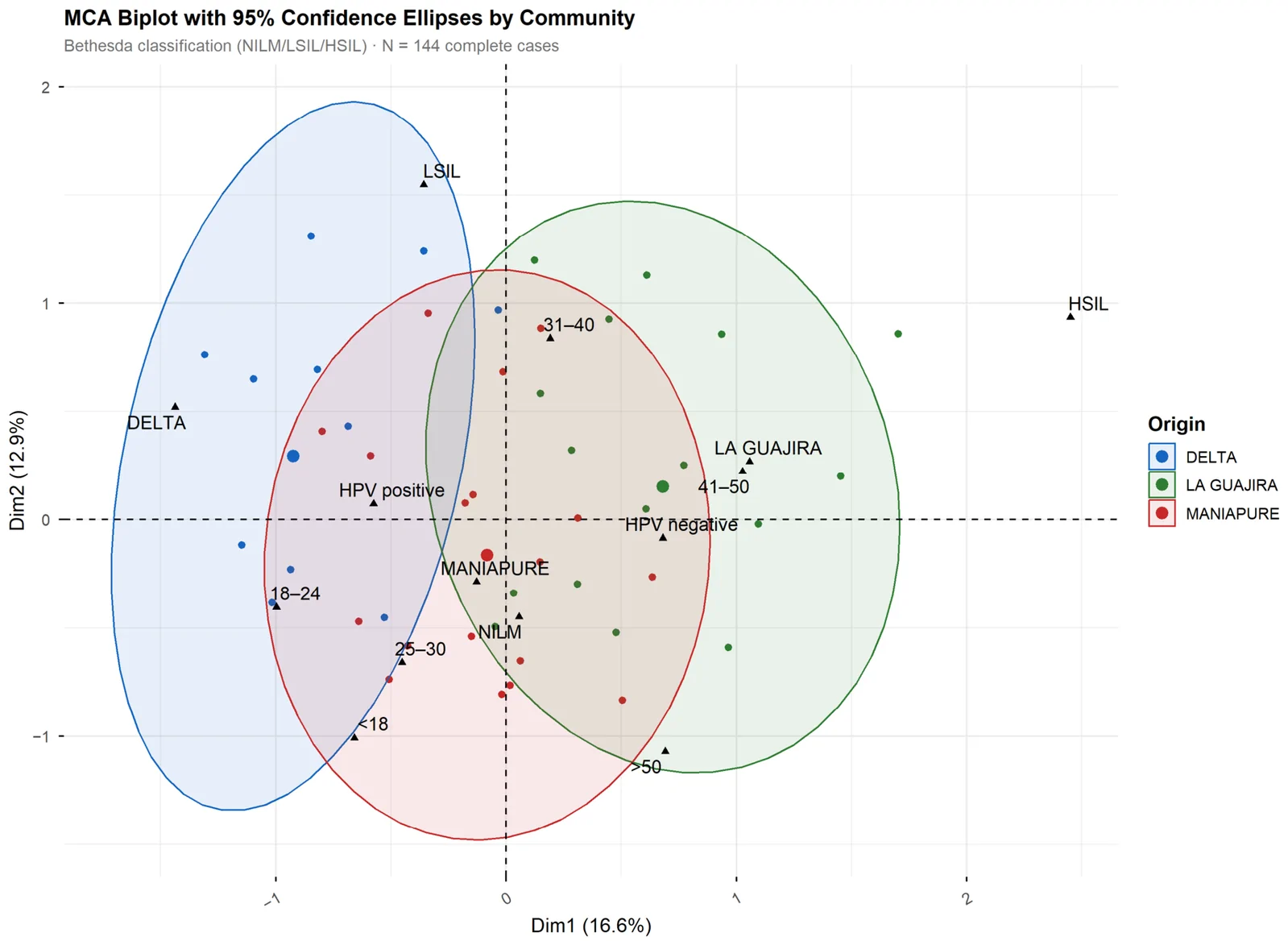
Multiple correspondence analysis of the 144 women with complete data (origin, age group, Bethesda category and HPV status), showing the first two dimensions with 95% confidence ellipses by community. Delta Amacuro and La Guajira occupy opposite ends of the first dimension, with Maniapure close to the centroid and overlapping both.

**Table 1:** General, cytological and virological characteristics of the study population, stratified by community. Percentages for cytological variables are calculated on the number of women with an available cytological result (overall *n* = 144; La Guajira *n* = 41; Maniapure *n* = 80; Delta Amacuro *n* = 23); all other percentages are calculated on the full analyzed sample. Additional findings within NILM are mutually exclusive and are given as percentages of women with NILM (overall *n* = 111; La Guajira *n* = 32; Maniapure *n* = 65; Delta Amacuro *n* = 14); “No additional findings” denotes NILM smears without inflammatory or infectious changes.

Variable	Overall (*n* = 260)	La Guajira (*n* = 94)	Maniapure (*n* = 141)	Delta Amacuro (*n* = 25)	*p*-Value	Test
Age, years, mean (SD)	34.0 (12.3)	37.7 (11.8)	33.0 (12.4)	25.6 (7.3)	<0.001	KW
Age, years, median (Q1–Q3)	32 (24–41)	38 (30–45)	31 (23–39)	25 (19–30)		
Cytology, risk scale, *n* (%)					0.047	Fisher
NILM	111 (77.1)	32 (78.0)	65 (81.2)	14 (60.9)		
LSIL	31 (21.5)	7 (17.1)	15 (18.8)	9 (39.1)		
HSIL	2 (1.4)	2 (4.9)	0 (0.0)	0 (0.0)		
NILM, additional findings (descriptive), *n* (% of NILM)					<0.001	Fisher
No additional findings	72 (64.9)	32 (100.0)	39 (60.0)	1 (7.1)		
Inflammatory changes	29 (26.1)	0 (0.0)	21 (32.3)	8 (57.1)		
Infectious changes	10 (9.0)	0 (0.0)	5 (7.7)	5 (35.7)		
HPV status by qPCR, *n* (%)					0.001	χ^2^
HPV-positive	144 (55.4)	40 (42.6)	84 (59.6)	20 (80.0)		
High-risk HPV-positive	133 (51.2)	37 (39.4)	76 (53.9)	20 (80.0)	<0.001	χ^2^
Coinfection pattern, *n* (%)					0.006	Fisher
No genotype detected	116 (44.6)	54 (57.4)	57 (40.4)	5 (20.0)		
1 genotype	70 (26.9)	25 (26.6)	42 (29.8)	3 (12.0)		
2 genotypes	39 (15.0)	7 (7.4)	25 (17.7)	7 (28.0)		
3 genotypes	16 (6.2)	5 (5.3)	8 (5.7)	3 (12.0)		
≥4 genotypes	19 (7.3)	3 (3.2)	9 (6.4)	7 (28.0)		

Table legends: KW: Kruskal–Wallis test; NILM: negative for intraepithelial lesion or malignancy; LSIL: low-grade squamous intraepithelial lesion; HSIL: high-grade squamous intraepithelial lesion; qPCR: real-time polymerase chain reaction; SD: standard deviation.

**Table 2:** HPV and high-risk HPV prevalence, overall and by community. Confidence intervals were calculated using the Wilson score method.

Group	*n* Tested	HPV-Positive, *n*	HPV Prevalence, % (95% CI)	High-Risk HPV-Positive, *n*	High-Risk Prevalence, % (95% CI)
**Overall**	260	144	55.4 (49.3–61.3)	133	51.2 (45.1–57.2)
La Guajira	94	40	42.6 (33.0–52.6)	37	39.4 (30.1–49.5)
Maniapure	141	84	59.6 (51.3–67.3)	76	53.9 (45.7–61.9)
Delta Amacuro	25	20	80.0 (60.9–91.1)	20	80.0 (60.9–91.1)

**Table 3:** HPV DNA detection and genotype distribution by cytological category among women with both results available. Each woman is counted once for every genotype she carried, so genotype rows do not sum to the column total.

Genotype	WHO 2026 Group	NILM (*n* = 111)	LSIL (*n* = 31)	*p*-Value
Any HPV detected		58 (52.3)	20 (64.5)	0.31
HPV-6	–	5 (4.5)	1 (3.2)	1.00
HPV-16	1a	14 (12.6)	3 (9.7)	1.00
HPV-18	1b	8 (7.2)	3 (9.7)	0.71
HPV-31	1c	14 (12.6)	8 (25.8)	0.09
HPV-33	1c	1 (0.9)	0 (0.0)	1.00
HPV-35	1c	2 (1.8)	1 (3.2)	0.53
HPV-39	1d	6 (5.4)	0 (0.0)	0.34
HPV-44	–	3 (2.7)	1 (3.2)	1.00
HPV-45	1b	6 (5.4)	3 (9.7)	0.41
HPV-51	1d	4 (3.6)	3 (9.7)	0.18
HPV-52	1c	8 (7.2)	1 (3.2)	0.68
HPV-53	–	9 (8.1)	1 (3.2)	0.69
HPV-56	1d	14 (12.6)	2 (6.5)	0.52
HPV-58	1c	5 (4.5)	0 (0.0)	0.59
HPV-59	1d	4 (3.6)	3 (9.7)	0.18
HPV-66	–	6 (5.4)	0 (0.0)	0.34
HPV-68	–	12 (10.8)	1 (3.2)	0.30
HPV-73	–	1 (0.9)	0 (0.0)	1.00
HPV-82	–	1 (0.9)	0 (0.0)	1.00

Table legend: Values are *n* (%) of all women in each cytological category. *p*-values from Fisher’s exact test, NILM versus LSIL. WHO 2026 group: risk group for management in the 2026 WHO guideline [44]; –, genotype not included in these groups. The two women with HSIL (both from La Guajira) had no HPV DNA detected and are not included in the table.

**Table 4:** Proportion of HPV-positive women carrying at least one genotype targeted by each licensed HPV vaccine formulation. A woman is counted once per formulation if any detected genotype is included in that formulation; categories are therefore not mutually exclusive. Values are *n*; % (95% CI, Wilson score method).

Formulation	Overall (*n* = 144)	La Guajira (*n* = 40)	Maniapure (*n* = 84)	Delta Amacuro (*n* = 20)
Bivalent (HPV-16/18)	44; 30.6 (23.6–38.5)	11; 27.5 (16.1–42.8)	23; 27.4 (19.0–37.7)	10; 50.0 (29.9–70.1)
Quadrivalent (HPV-6/11/16/18)	53; 36.8 (29.4–44.9)	12; 30.0 (18.1–45.4)	29; 34.5 (25.2–45.2)	12; 60.0 (38.7–78.1)
Nonavalent (HPV-6/11/16/18/31/33/45/52/58)	100; 69.4 (61.5–76.4)	23; 57.5 (42.2–71.5)	59; 70.2 (59.8–79.0)	18; 90.0 (69.9–97.2)
No genotype targeted by any formulation	44; 30.6 (23.6–38.5)	17; 42.5 (28.5–57.8)	25; 29.8 (21.0–40.2)	2; 10.0 (2.8–30.1)

## Data Availability

The data are not publicly available due to privacy and ethical restrictions protecting the participating indigenous communities. A written request with reasonable justification may be sent to the corresponding author.

## Supplementary Materials

The following supporting information can be downloaded at: https://www.mdpi.com/article/10.3390/diseases14100384/s1, Supplementary Methods S1: HPV genotyping assay, quality control and contamination prevention; Table S1: Age by community: descriptive statistics, Kruskal–Wallis test and Dunn post hoc comparisons (Bonferroni correction); Table S2: Age by HPV status; Table S3: Comparisons of proportions between communities; Table S4: Cytological findings by community, among women with cytology; Table S5: Cytological findings by HPV status, among women with both results; Figure S1: Prevalence of any HPV and of high-risk HPV, overall and by community, with 95% Wilson confidence intervals; Table S6: High-risk HPV prevalence under three classifications, overall and by community; Table S7: HPV prevalence by age group, overall and by community; Figure S2: HPV positivity by age group and community; Table S8: Five most frequent HPV genotypes by age group; Table S9: Distribution of the 19 HPV genotypes detected, overall and by community; Table S10: HPV-positive women by the highest-risk WHO 2026 management group among their genotypes, overall and by community; Figure S3: Distribution by WHO 2026 management group and community; Figure S4: Number of concurrent HPV genotypes among HPV-positive women, overall and by community; Table S11: Cross-tabulation of conventional cytology and qPCR results, with Cohen’s κ, overall and by community; Table S12: Vaccine-targeted genotypes at woman level and infection level; Figure S5: Proportion of HPV-positive women carrying at least one genotype included in each licensed vaccine formulation, overall and by community; Table S13: Multiple correspondence analysis: variance explained by dimension; Table S14: Multiple correspondence analysis: coordinates, contributions and cos^2^ of each category on the first two dimensions; Figure S6: Contribution of each category to the first two dimensions of the multiple correspondence analysis.

## Abbreviations

CI: confidence interval; CIN: cervical intraepithelial neoplasia; HPV: human papillomavirus; hrHPV: high-risk human papillomavirus; HSIL: high-grade squamous intraepithelial lesion; IARC: International Agency for Research on Cancer; LSIL: low-grade squamous intraepithelial lesion; MCA: multiple correspondence analysis; NILM: negative for intraepithelial lesion or malignancy; PCR: polymerase chain reaction; qPCR: real-time polymerase chain reaction; WHO: World Health Organization; κ: Cohen’s kappa coefficient.
