# Supplementary material for: Divergent Human Papillomavirus Genotype Spectra in Three Indigenous Communities of Venezuela: Implications for Vaccine Choice and Cervical Screening
Source: Diseases. 2026 Oct 19;14(10):384. doi: 10.3390/diseases14100384 (PMC13703561; doi:10.3390/diseases14100384)
Supplement: Supplementary file 1 [file diseases-14-00384-s001.zip › diseases-4574419-supplementary.pdf]

Supplementary Materials

Divergent Human Papillomavirus Genotype Spectra in Three Indigenous Communities of Venezuela: Implications for Vaccine Choice and Cervical Screening

Supplementary Methods S1

S1.1. HPV genotyping assay

HPV detection and genotyping used the HPV Genotypes 21 Real-TM Quant kit (Sacace Biotechnologies, Como, Italy; REF V21-100 FRT; CE-IVD) on a Bio-Rad CFX Opus 96 real-time PCR system. The assay resolves 21 HPV types individually (HPV-6, 11, 16, 18, 26, 31, 33, 35, 39, 44, 45, 51, 52, 53, 56, 58, 59, 66, 68, 73 and 82) in eight reaction tubes per sample. Tubes 1 to 7 each detect three genotypes (FAM, ROX and Cy5 channels) together with an internal control (HEX channel). Tube 8 contains a sample intake control that amplifies human genomic DNA (FAM channel) to check cellular adequacy, plus the internal control and a strip orientation marker.

The assay uses hydrolysis (TaqMan) probes with a paraffin-wax hot start. Its analytical limit of detection is 5 copies of viral DNA per reaction (10^3 copies/mL of extracted sample). A genotype was called positive when its channel showed an exponential amplification curve crossing the threshold, following the manufacturer's instructions. The manufacturer reports 100% analytical specificity, with no cross-reactivity against common urogenital pathogens (including *Chlamydia trachomatis*, *Neisseria gonorrhoeae*, *Trichomonas vaginalis*, *Mycoplasma* and *Ureaplasma* spp., herpesviruses and *Candida* spp.), and a diagnostic sensitivity of 99.3% (95% CI 96.7–100) with a specificity of 99.9% (95% CI 99.8–99.9). Primer and probe sequences are proprietary and are not disclosed by the manufacturer.

The manufacturer groups 18 of the 21 types as high-risk or potentially high-risk, including HPV-26, 53, 73 and 82. For consistency with IARC and WHO, the main analysis used the narrower definition of 14 high-risk types described in Section 2.6; the alternative definitions are compared in Table S6.

S1.2. Contamination control

Sample handling and DNA extraction, in a biosafety cabinet, and the PCR stage were carried out in physically separate areas with a one-way workflow, dedicated pipettes, aerosol-barrier filter tips and laboratory coats. Each extraction batch included a blank extraction control (sterile saline) taken through the whole procedure, and each PCR run included a no-template control and the manufacturer's multi-genotype positive control. A run was accepted only if both negative controls showed no amplification in any channel; otherwise it was repeated after decontamination. No negative control amplified during the study, and every sample was tested in two separate PCR runs. Each genotype is read through its own probe and channel combination, so the coinfections reported reflect separate type-specific signals.

S1.3. Sample storage and integrity

Swabs were placed in a nucleic-acid-stabilizing lysis buffer that lyses cells and inactivates nucleases at once, protecting DNA without refrigeration. Samples were transported under refrigeration and stored at −20 °C upon arrival in Caracas. Once all samples from a community were received, we thawed and processed them together, so DNA extraction and PCR occurred in batches, one community at a time, 1 to 3 months after collection. We checked DNA integrity by electrophoresis on 0.75% agarose gels, and we checked every sample with the sample intake control (which flagged degraded or insufficient material) and the internal control in all eight tubes (which showed inhibition). DNA yield was 50–100 µg/mL.

Supplementary Tables and Figures

Table S1. Age by community: descriptive statistics, Kruskal–Wallis test and Dunn post hoc comparisons (Bonferroni correction).

Community	n	Mean ± SD (years)	Median (Q1–Q3)
Delta Amacuro	25	25.6 ± 7.3	25 (19.0–30.0)
La Guajira	94	37.7 ± 11.8	38 (30.2–44.8)
Maniapure	141	33.0 ± 12.4	31 (23.0–39.0)
Kruskal–Wallis		H = 27.44, df = 2	$p < 0.001$
Delta Amacuro vs. La Guajira		Dunn, adjusted	$p < 0.001$
Delta Amacuro vs. Maniapure		Dunn, adjusted	$p = 0.005$
La Guajira vs. Maniapure		Dunn, adjusted	$p = 0.001$

Age was not normally distributed (Shapiro–Wilk $p < 0.001$ in Maniapure); Levene test $p = 0.079$.

Table S2. Age by HPV status.

HPV status	n	Mean ± SD (years)	Median (Q1–Q3)
HPV-negative	116	36.7 ± 11.4	38 (28.8–44.0)
HPV-positive	144	31.8 ± 12.5	30 (22.0–38.0)
Mann–Whitney U		W = 10849	$p < 0.001$

Mean difference 4.9 years (95% CI 1.96–7.82).

Table S3. Comparisons of proportions between communities.

Variable	Test	Statistic	p-value
HPV status	Pearson χ^2	$\chi^2 = 13.40$, df = 2	0.001
High-risk HPV status	Pearson χ^2	$\chi^2 = 13.98$, df = 2	< 0.001
Cytology (NILM, LSIL, HSIL)	Fisher's exact	–	0.047
Number of genotypes, HPV-positive women (categories)	Fisher's exact	–	0.006
Number of genotypes per HPV-positive woman	Kruskal–Wallis	H = 13.87, df = 2	< 0.001

Fisher's exact tests used a simulated p-value (B = 10,000) when expected counts were small. The cytology comparison rests on only two HSIL cases and should be read with caution.

Table S4. Cytological findings by community, among women with cytology (descriptive).

Finding	Delta Amacuro (n = 23)	La Guajira (n = 41)	Maniapure (n = 80)
NILM, no other finding	1 (4.3)	32 (78.0)	39 (48.8)
NILM, inflammatory changes	8 (34.8)	0 (0.0)	21 (26.2)
NILM, bacterial vaginosis	2 (8.7)	0 (0.0)	4 (5.0)
NILM, Candida spp.	2 (8.7)	0 (0.0)	1 (1.2)
NILM, Trichomonas spp.	1 (4.3)	0 (0.0)	0 (0.0)
LSIL or HSIL	9 (39.1)	9 (22.0)	15 (18.8)

Values are n (%). Inflammatory and infectious changes fall within the NILM category and are reported descriptively only.

Table S5. Cytological findings by HPV status, among women with both results (descriptive).

Finding	HPV-negative (n = 66)	HPV-positive (n = 78)
NILM, no other finding	35 (53.0)	37 (47.4)
NILM, inflammatory changes	15 (22.7)	14 (17.9)
NILM, bacterial vaginosis	2 (3.0)	4 (5.1)
NILM, Candida spp.	1 (1.5)	2 (2.6)
NILM, Trichomonas spp.	0 (0.0)	1 (1.3)
LSIL or HSIL	13 (19.7)	20 (25.6)

Values are n (%).

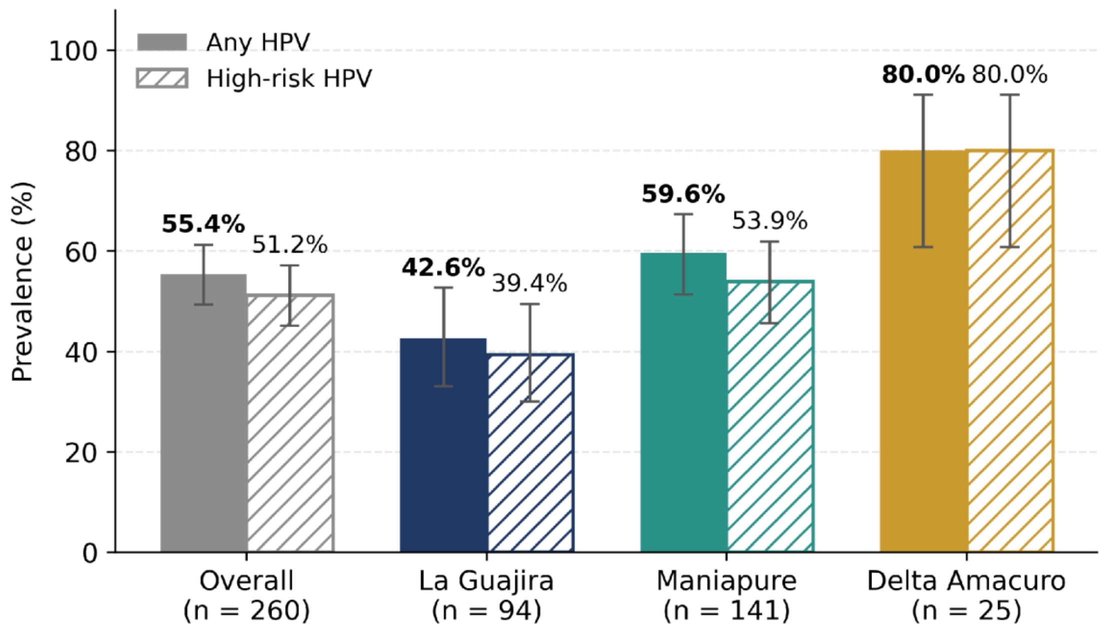

Figure S1. Prevalence of any HPV and of high-risk HPV, overall and by community, with 95% Wilson confidence intervals. In Delta Amacuro, every HPV-positive woman carried at least one high-risk genotype, so the two estimates coincide.

Table S6. High-risk HPV prevalence under three classifications, overall and by community.

Community	n	IARC group 1 (12 types)	Study definition (14 types)	Manufacturer (17 types detected)
Delta Amacuro	25	20 (80.0)	20 (80.0)	20 (80.0)
La Guajira	94	34 (36.2)	37 (39.4)	40 (42.6)
Maniapure	141	73 (51.8)	76 (53.9)	79 (56.0)
Overall	260	127 (48.8)	133 (51.2)	139 (53.5)

Values are n (%). Study definition: IARC group 1 plus HPV-68 (group 2A) and HPV-66 (group 2B). The manufacturer's definition adds HPV-53, 73 and 82; HPV-26, also included by the manufacturer, was not detected.

Table S7. HPV prevalence by age group, overall and by community.

Age group (years)	Overall	La Guajira	Maniapure	Delta Amacuro
<18	5/9 (55.6)	1/2 (50.0)	3/5 (60.0)	1/2 (50.0)
18–24	44/58 (75.9)	7/13 (53.8)	29/36 (80.6)	8/9 (88.9)
25–30	28/43 (65.1)	7/9 (77.8)	14/26 (53.8)	7/8 (87.5)
31–40	43/81 (53.1)	14/32 (43.8)	25/44 (56.8)	4/5 (80.0)
41–50	11/45 (24.4)	6/26 (23.1)	5/18 (27.8)	0/1 (0.0)
>50	13/24 (54.2)	5/12 (41.7)	8/12 (66.7)	–

Values are HPV-positive/tested (%). –, no women in this group. Several cells are small and estimates are descriptive.

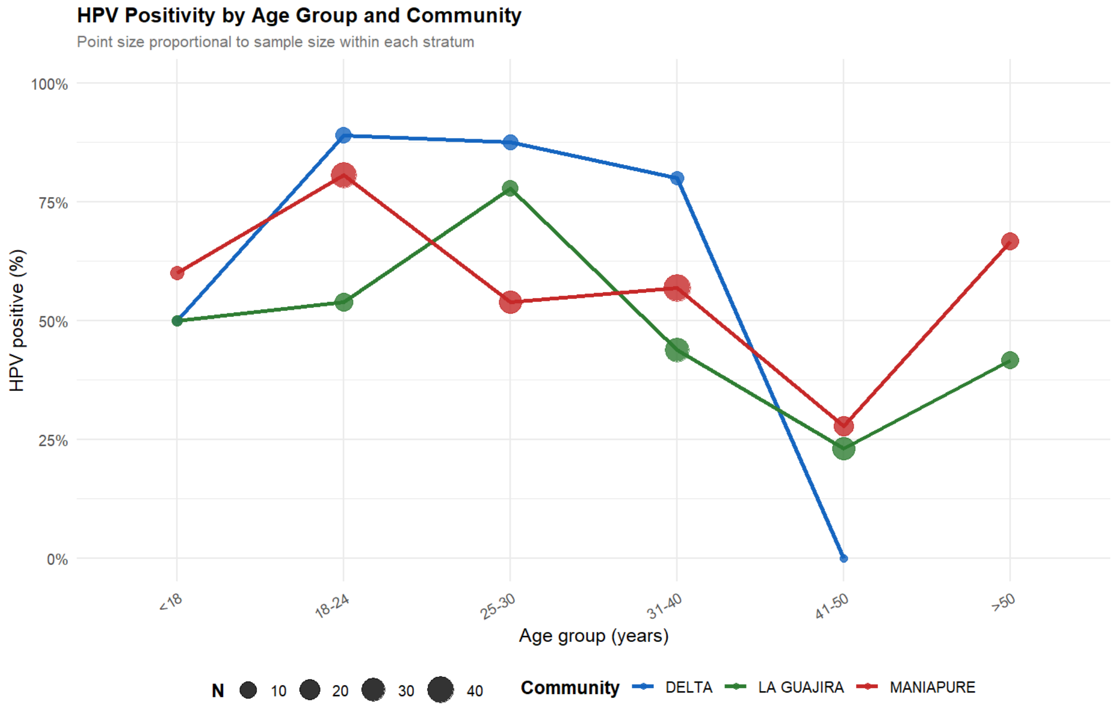

Figure S2. HPV positivity by age group and community. Point size is proportional to the number of women in each stratum.

Table S8. Five most frequent HPV genotypes by age group.

Age group (years)	Most frequent genotypes, % of women tested
<18 (n = 9)	HPV-16 (33.3), HPV-59 (33.3), HPV-53 (22.2), HPV-56 (22.2), HPV-18 (11.1)
18–24 (n = 58)	HPV-31 (22.4), HPV-18 (19.0), HPV-16 (15.5), HPV-53 (13.8), HPV-68 (13.8)
25–30 (n = 43)	HPV-31 (20.9), HPV-16 (16.3), HPV-56 (16.3), HPV-18 (14.0), HPV-51 (14.0)
31–40 (n = 81)	HPV-31 (16.0), HPV-58 (11.1), HPV-16 (9.9), HPV-45 (8.6), HPV-39 (7.4)
41–50 (n = 45)	HPV-31 (8.9), HPV-18 (4.4), HPV-59 (4.4), HPV-66 (4.4), HPV-68 (4.4)
>50 (n = 24)	HPV-52 (20.8), HPV-39 (16.7), HPV-51 (12.5), HPV-59 (12.5), HPV-66 (12.5)

Table S9. Distribution of the 19 HPV genotypes detected, overall and by community.

Genotype	Risk category	Overall (n = 260)	La Guajira (n = 94)	Maniapure (n = 141)	Delta Amacuro (n = 25)
HPV-31	High-risk	41 (15.8)	3 (3.2)	29 (20.6)	9 (36.0)
HPV-16	High-risk	30 (11.5)	4 (4.3)	17 (12.1)	9 (36.0)
HPV-18	High-risk	24 (9.2)	8 (8.5)	13 (9.2)	3 (12.0)
HPV-56	High-risk	22 (8.5)	3 (3.2)	9 (6.4)	10 (40.0)
HPV-39	High-risk	21 (8.1)	4 (4.3)	16 (11.3)	1 (4.0)
HPV-45	High-risk	19 (7.3)	6 (6.4)	8 (5.7)	5 (20.0)
HPV-51	High-risk	19 (7.3)	9 (9.6)	7 (5.0)	3 (12.0)
HPV-68	High-risk	18 (6.9)	5 (5.3)	1 (0.7)	12 (48.0)
HPV-59	High-risk	17 (6.5)	6 (6.4)	10 (7.1)	1 (4.0)
HPV-53	Possibly carcinogenic	15 (5.8)	2 (2.1)	11 (7.8)	2 (8.0)

Genotype	Risk category	Overall (n = 260)	La Guajira (n = 94)	Maniapure (n = 141)	Delta Amacuro (n = 25)
HPV-52	High-risk	15 (5.8)	3 (3.2)	12 (8.5)	0 (0.0)
HPV-58	High-risk	14 (5.4)	4 (4.3)	9 (6.4)	1 (4.0)
HPV-6	Low-risk	10 (3.8)	1 (1.1)	7 (5.0)	2 (8.0)
HPV-66	High-risk	10 (3.8)	4 (4.3)	6 (4.3)	0 (0.0)
HPV-44	Low-risk	6 (2.3)	2 (2.1)	3 (2.1)	1 (4.0)
HPV-82	Possibly carcinogenic	4 (1.5)	2 (2.1)	1 (0.7)	1 (4.0)
HPV-35	High-risk	4 (1.5)	3 (3.2)	1 (0.7)	0 (0.0)
HPV-73	Possibly carcinogenic	2 (0.8)	2 (2.1)	0 (0.0)	0 (0.0)
HPV-33	High-risk	1 (0.4)	0 (0.0)	1 (0.7)	0 (0.0)

Values are n (%) of women tested in whom each genotype was detected; because coinfections are frequent, column percentages sum to more than the corresponding HPV prevalence. Risk category follows IARC; possibly carcinogenic, IARC group 2B.

Table S10. HPV-positive women by the highest-risk WHO 2026 management group among their genotypes, overall and by community.

WHO 2026 group (highest-risk type)	Overall (n = 144)	La Guajira (n = 40)	Maniapure (n = 84)	Delta Amacuro (n = 20)
1a (HPV-16)	30 (20.8)	4 (10.0)	17 (20.2)	9 (45.0)
1b (HPV-18, 45)	26 (18.1)	11 (27.5)	10 (11.9)	5 (25.0)
1c (HPV-31, 33, 35, 52, 58)	39 (27.1)	9 (22.5)	28 (33.3)	2 (10.0)
1d (HPV-39, 51, 56, 59)	32 (22.2)	10 (25.0)	18 (21.4)	4 (20.0)
Only types outside the WHO groups	17 (11.8)	6 (15.0)	11 (13.1)	0 (0.0)

Values are n (%) of HPV-positive women, each counted once in the highest-risk group among the types she carried. Types outside the groups: HPV-6, 44, 53, 66, 68, 73 and 82.

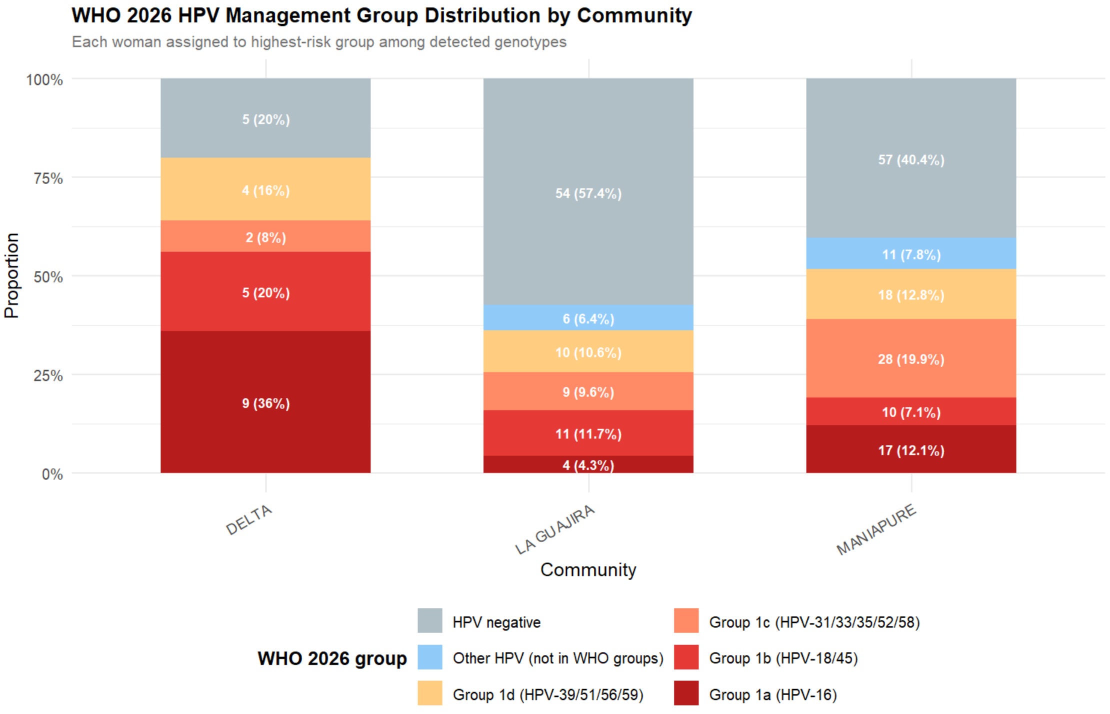

Figure S3. Distribution by WHO 2026 management group and community.

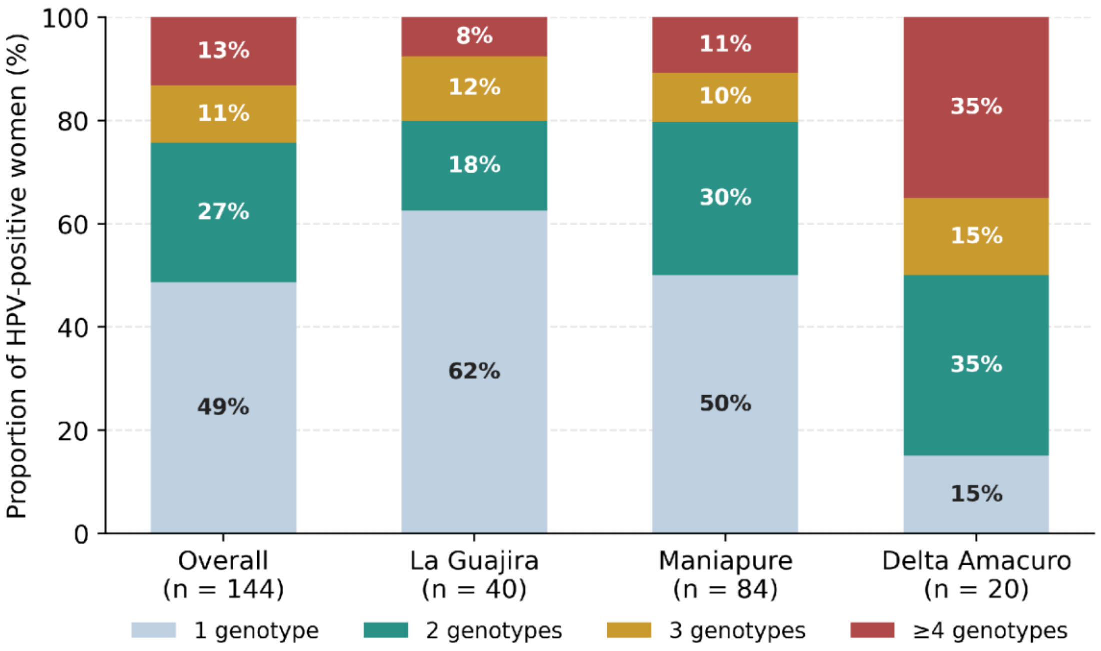

Figure S4. Number of concurrent HPV genotypes among HPV-positive women, overall and by community. Percentages are calculated on HPV-positive women only.

Table S11. Cross-tabulation of conventional cytology and qPCR results, with Cohen's κ, overall and by community.

Community	n	HPV+/Cyt+	HPV+/Cyt–	HPV–/Cyt+	HPV–/Cyt–	Cohen's κ
Overall	144	20	58	13	53	0.056
La Guajira	41	4	13	5	19	0.029

Community	n	HPV+/Cyt+	HPV+/Cyt−	HPV−/Cyt+	HPV−/Cyt−	Cohen's κ
Maniapure	80	10	32	5	33	0.103
Delta Amacuro	23	6	13	3	1	−0.219

Cyt+, LSIL or HSIL; Cyt−, NILM. Neither test is treated as the reference standard, and no sensitivity, specificity or predictive values are reported. κ interpreted according to Landis and Koch.

Table S12. Vaccine-targeted genotypes at woman level and infection level, % (95% CI).

Formulation	Woman level (n = 144)	All detections (n = 292)	High-risk detections (n = 255)
Bivalent (HPV-16, 18)	30.6 (23.6–38.5)	18.5 (14.5–23.3)	21.2 (16.6–26.6)
Quadrivalent (HPV-6, 11, 16, 18)	36.8 (29.4–44.9)	21.9 (17.6–27.0)	21.2 (16.6–26.6)
Nonavalent (adds HPV-31, 33, 45, 52, 58)	69.4 (61.5–76.4)	52.7 (47.0–58.4)	56.5 (50.3–62.4)

Wilson 95% confidence intervals. Infection-level intervals ignore clustering of several genotypes within the same woman and are approximate. HPV-11 was included in the assay and not detected in any sample, so these estimates are complete.

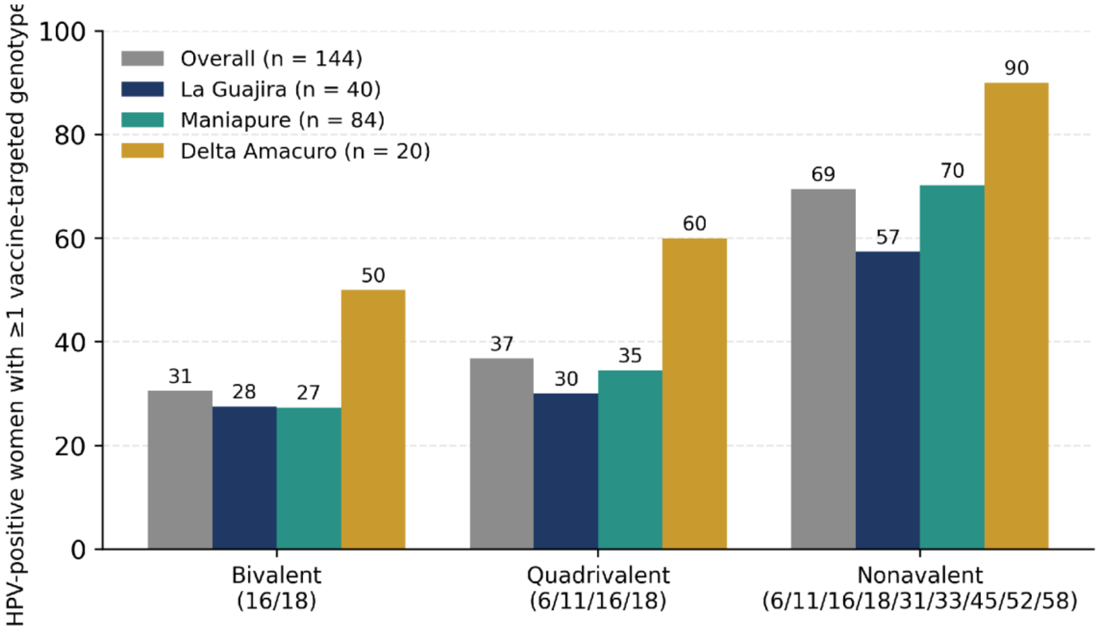

Figure S5. Proportion of HPV-positive women carrying at least one genotype included in each licensed vaccine formulation, overall and by community.

Table S13. Multiple correspondence analysis: variance explained by dimension.

Dimension	Eigenvalue	Variance (%)	Cumulative (%)
1	0.41	16.56	16.56
2	0.32	12.90	29.46
3	0.29	11.68	41.14
4	0.28	11.14	52.28
5	0.26	10.40	62.68

Table S14. Multiple correspondence analysis: coordinates, contributions (%) and cos² of each category on the first two dimensions.

Category	Dim 1 coord.	Dim 2 coord.	Dim 1 contrib.	Dim 2 contrib.	Dim 1 cos²	Dim 2 cos²
Delta Amacuro	−1.437	0.519	19.92	3.34	0.393	0.051
La Guajira	1.057	0.267	19.20	1.58	0.444	0.028
Maniapure	−0.128	−0.286	0.55	3.53	0.021	0.102
<18 years	−0.659	−1.009	0.91	2.74	0.016	0.037
18–24 years	−0.997	−0.404	12.93	2.72	0.273	0.045
25–30 years	−0.452	−0.660	1.80	4.92	0.035	0.074
31–40 years	0.192	0.838	0.76	18.53	0.019	0.363
41–50 years	1.026	0.222	10.60	0.64	0.211	0.010
>50 years	0.691	−1.071	2.80	8.64	0.051	0.124
NILM	0.056	−0.449	0.14	12.03	0.010	0.678
LSIL	−0.358	1.547	1.67	39.92	0.035	0.656
HSIL	2.451	0.935	5.04	0.94	0.085	0.012
HPV-negative	0.681	−0.085	12.82	0.26	0.392	0.006
HPV-positive	−0.576	0.072	10.85	0.22	0.392	0.006

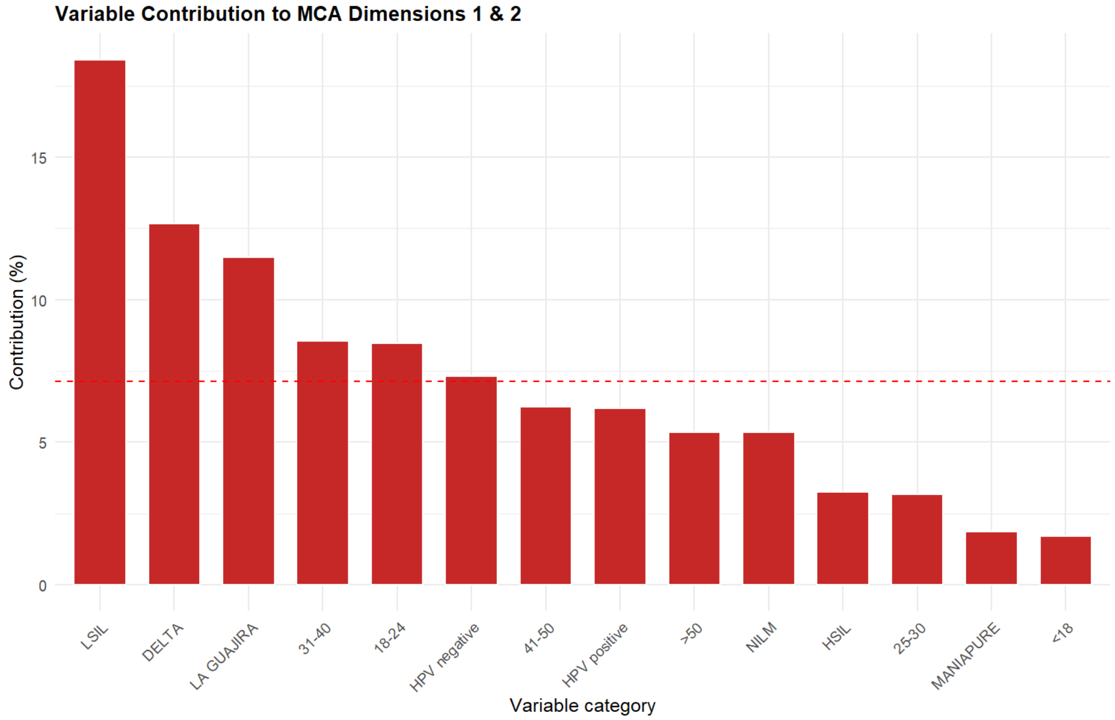

Figure S6. Contribution of each category to the first two dimensions of the multiple correspondence analysis. The dashed line marks the contribution expected if all categories contributed equally.
